# A Water-Soluble Core for Manufacturing Hollow Injection-Molded Products

**DOI:** 10.3390/polym14112185

**Published:** 2022-05-27

**Authors:** Chung-Chih Lin, Chao-Long Yang

**Affiliations:** Department of Mechanical and Computer-Aided Engineering, National Formosa University, No. 64, Wen-Hwa Road, Hu-Wei Town, Yunlin County 632003, Taiwan; linccnfu@gmail.com

**Keywords:** injection molding, water-soluble core, insert molding, Taguchi method

## Abstract

To manufacture a complicated hollow product without any assembly process, for example, the plastic intake manifold, is difficult by the traditional injection molding method. The fusible-core technique, which uses a low-melting-point alloy as a sacrificial core, was developed to solve this problem; however, the limited selection of resin type and the huge capital investment have caused this technique to spread slowly. In this work, a novel method is established that can produce similar products without the limitation of resin type, as well as a lower-energy-consumption process. The concept of the enveloped core defined by a water-soluble core assembled with a shell is proposed herein; it provides both rigidity and toughness to resist the pressure during the injection molding process. The shape of the enveloped core equals the internal contour of the designated product. An insert molding process was introduced to cover the enveloped core with a skin layer. Cut out the end of the enveloped core and immerse it into a water bath. When the water-soluble core inside the shell is dissolved, the product with a special internal contour is accomplished. A tee-joint is presented to demonstrate how the proposed method can be utilized. The optimal ingredient of the core and processing parameters are determined by the Taguchi method. The result shows that the proposed product is molded successfully when the compressive strength of the core is larger than 2 MPa. In addition, the eccentricity measurement of internal contour of the optimal sample exhibits a 56% improvement, and the required time for the core removal is less than 154 s.

## 1. Introduction

Two plastic molding methods, extrusion molding and injection molding, provide the main approaches for mass production in the plastic industry. In general, a hollow product with a simple profile, e.g., pipe or bottle, is usually produced by the extrusion or extrusion-blow method [1,2,3]. However, when a hollow product has a complicated internal contour, e.g., the plastic intake manifold of a car engine, it will be difficult to be molded by the extrusion method [4]. Traditionally, the plastic intake manifold is usually divided into several subcomponents made individually by the injection method, and then an assembly process, such as mechanical fastening or bonding by adhesive, is used. The various injection molds to manufacture those subcomponents, and labor or post-processing to complete the product assembly, could increase the costs of manufacture and accumulate many dimensional errors [5,6]. Furthermore, the locations in which assembly processes are implemented, such as bonding or fastening, can be recognized as the weakest regions for the product, thus increasing the tendency of the product to fail in the case of high pressure or heavy loading operation. Moreover, the chemical solvents used frequently in the bonding process also contaminate the environment.

Removable or collapse core skill is commonly used for manufacturing hollow products, for example, sand cores for casting metallic exhaust manifold products [7]. Polymer cores such as polyoxymethylene (POM) or polystyrene (PS) foam are frequently used in resin transfer molding (RTM). When the resin is cured, those cores can be melted out or burned off at elevated temperatures [8,9]. Due to good solubility in water, the core made of salt to manufacture hollow composites is also welcome [7,8,9,10,11]. However, the pressure induced by the RTM process is much lower than that in the plastic injection process; this means that the brittle characteristic of the salt core cannot resist the pressure from cracking during the injection process. A highly viscous melt in the filling stage of the injection process also frequently causes the core shift problem. The fusible-core technique [12] provides an improved approach to manufacture a plastic product with a complicated internal geometry. This method involves the use of a low-melting-point alloy as a sacrificial core to be overmolded with a polymer (the skin layer). Afterward, the core is melted out inductively, using electricity, in a liquid heat-transfer medium. The metallic core with a high strength property gives the molded product good dimensional accuracy. However, the selection of plastic types for this method is limited, and only those high-performance engineering plastics are suitable. In addition, the high energy consumption and the huge capital investment cause this method to spread slowly. McNulty et al. [13] developed another method for creating internal features by using water-soluble polyvinyl alcohol (PVOH) patterns in the injection process. Most of the area of the soluble pattern is supported by the mold cavity, which differs from the fusible-core technique used for hollow product manufacture. Although these proposed methods are used in different applications, there is always room for improvement, especially in the field of injection molding to produce a special hollow product.

In this work, the concept of an enveloped core defined by a water-soluble core assembled with a shell is proposed. The water-soluble core consists of NaCl (salt) and trehalose sugar (binder), both of which are water-soluble. When proper temperature and pressure are simultaneously applied to the mixture, both the NaCl and trehalose sugar can glue together and achieve adequate rigidity. The shell is made of plastic. The cooperation of the core and the shell makes it not only rigid, but also tough to resist the deformation induced by injection pressure. The optimal ingredient of the core and processing parameters were determined by using the Taguchi method. A tee-joint product is proposed to demonstrate how the method is to be executed. The influence of the binder content, applied pressure, processing temperature, and processing time on the core strength and the dimension accuracy of the molded product, as well as the core removal rate, are all investigated.

## 2. Fabrication Process and Product Design

### 2.1. Fabrication Process

**Figure 1:**
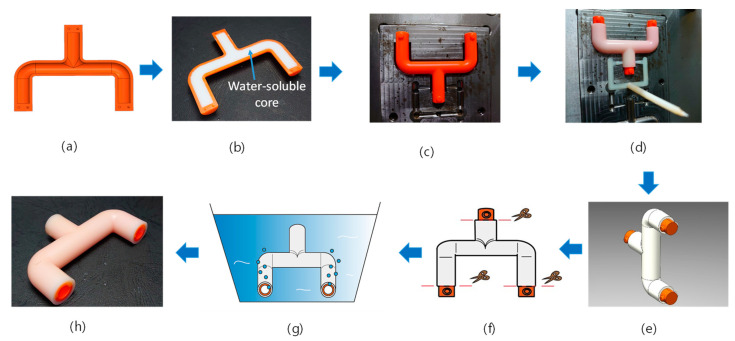
Preparation procedures to produce a hollow product by using water-soluble core and (**a**) shell (**b**): the water-soluble core with a shell is prepared; (**c**) the enveloped core is prepared by two core shells and located in the mold cavity, (**d**) the skin layer is molded, (**e**) the product ejected from the mold, (**f**) the ends are cut out of the enveloped core, (**g**) the product is immersed into a water bath and core dissolution, and (**h**) the final product is obtained.

The fabrication process is described as a water-soluble core, representing a half internal contour of the designated product prepared in advance. A shell used to protect the core is made by injection or other methods, as shown in Figure 1a. The core is put into the shell and then becomes a core–shell, as shown in Figure 1b. Assemble two core–shells into an enveloped core and locate it in the mold cavity, as shown in Figure 1c. Afterward, the skin layer is molded by an insert molding process, and the molded product is accomplished, as shown in Figure 1d,e. Consequently, three ends of the enveloped core appear outside the skin layer and are cut out, and the product is immersed into a water bath, as shown in Figure 1f,g. When the filler inside the core is dissolved, the final product is completed, as shown in Figure 1h.

### 2.2. Design Guidance of the Shell Model and the Skin Layer Model of the Product

**Figure 2:**
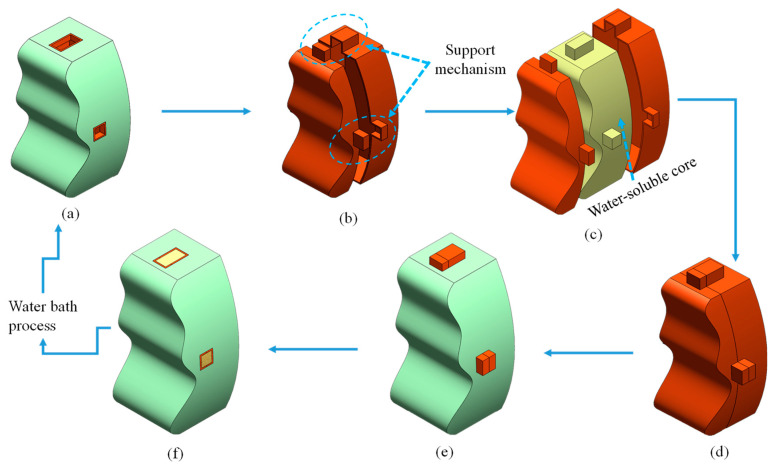
Schematic illustration of design flow: (**a**) the designated product, (**b**) two halves of the shell model generated from the internal contour of the product, (**c**) the sketch of the core assembled into the shells, (**d**) the enveloped core, (**e**) the skin model covers the core entirely, and (**f**) cut out the support mechanism.

When a product is to be produced by this method, two related design models, the shell model and the skin layer model, need to be generated in accordance with the manufacturability consideration. Taking a designated product illustrated in Figure 2a as an example, the green color and the orange color represent the shell model and the skin layer model of the product, respectively. The arrow line shows the design process. The geometry of the shell model shown in Figure 2b was extracted from the internal contour of the designated product and divided into two halves for the core assembly. Note that some additional support mechanisms are generated and integrated into the shell model. In general, the number of the orifice in the product equals the number of the support mechanisms. The inner space constructed by both halves of the shell model shown in Figure 2c equals the volume of the core. The enveloped core shown in Figure 2d represents the core assembled within the shell. The support mechanism correctly locates the enveloped core in the mold cavity, reducing the enveloped core’s shift during the injection molding process. The skin layer model (green color) shown in Figure 2e is the same as the designated product, except for the thickness. After an insert molding is executed, the enveloped core is covered by the skin layer. Cut out the support mechanism, and the final product is accomplished as the core is dissolved.

The enveloped core is entirely surrounded by the skin layer, and the separation between them is less. However, if the material-type selections for both models are the same, the adhesive strength is enhanced, and it also benefits the recyclable consideration. The thickness of both models depends on the product design requirement. If the product is used in heavy loading, the thickness of the skin layer model must be designed to be as large as possible. The thickness designs for both models also need to consider the strength and molding feasibility.

## 3. Water-Soluble Core Development

Salt is a mineral composed essentially of sodium chloride (NaCl), an inorganic compound with excellent solubility in water. The use of a salt core is known as the candidate for lost core molding, for example, die-casting processes or an RTM process [14,15]. In the major preparation of the salt core, the shooting method is welcome due to the low energy consumption of the preparation process and ease in molding a complicated core shape [11,16]. However, the brittle characteristic makes the salt core difficult to be used directly in the injection molding. The shear effect induced by a highly viscous melt flow during the injection process frequently makes the salt core fracture. Using the shell enhances the toughness of the core; however, the optimal ingredient of the fillers (salt/binder) and the processing parameters (temperature and pressure) for core preparation still need to be addressed.

The mixture used for the core contains salt (NaCl powder) and binder (trehalose sugar). The NaCl powder was derived from Taiyen Biotech Co., Ltd. (Taipei, Taiwan), and its particle size was in the range of 0.5–0.8 mm. Trehalose sugar, whose particle size was in the range of 0.3–0.50 mm, was supplied by Hayashibara Co., Ltd. (Okayama, Japan). Trehalose sugar is a nonreducing sugar that is formed from two glucose units joined by a 1-1 alpha bond [17,18]. It is therefore water-soluble and compatible with NaCl. Two powders, NaCl and Trehalose sugar, with a specified ratio were adequately blended and filled into the mold cavity of the salt core. When pressure loading is applied on the salt core and the mold put into an oven simultaneously, the solidification process of the mixture occurs. Using a binder to glue the salt can significantly reduce the required applied pressure; thus, a higher level of mechanical interlocking between the salt grains is not required to achieve an acceptable strength [11]. After the processing time is completed, the salt core is taken out of the mold, and a compression test investigating the effect of those parameters on compressive strength of the salt core is implemented.

### 3.1. Design of the Experiment

A good salt core provides sufficient compressive strength to resist the injection pressure. A Taguchi method is introduced to estimate the relative significance of a range of processing variables of the salt core. The setting variables and their associated levels are listed in Table 1. The processing temperature melts the trehalose sugar, which functions as a binder to effectively make the mixture consolidation. Some researchers [11,18,19] have revealed that the recommended processing temperature of trehalose sugar ranges from 120 to 190 °C. Tests were implemented by using Taguchi’s L_9_(3^4^) orthogonal array. The values of level 2 are determined based on the conventional value of each parameter. The values of level 1 and level 3 are the lower and higher values setting parameters, respectively. In this study, we used the *S*/*N* ratio to qualify the quality characteristic deviating from the designated aim; the larger-is-better approach was used for maximizing the compressive strength of the salt core. The formula of *S*/*N* ratio was expressed as follows:

(1) S/N=−10log101n∑i=1n1yi2

where *y_i_* is the *i*th measured compressive strength, and *n* is the total number of specimens in each trial. In addition to the *S*/*N* ratio, ANOVA was also employed to obtain the effect of the process parameters on mechanical properties.

### 3.2. Specimen Preparation and Characteristics

A compression test was conducted by referring to ASTM D695-15 and C473-15 standards for the compressive strength evaluation of the salt core. The specimen was designed as a cylinder with the following dimensions: 35 mm× 35 mm in diameter and height, respectively. The amount of the specimen made by each parameter combination is five. The test specimens were first conditioned for 24 h at 25 °C and 40% relative humidity before the compression test. The compression test was conducted on the HongTa testing machine (HT-2402, Taichung, Taiwan), at room temperature, i.e., 27 °C. A compression speed of 5 mm/min, referring to the standards, was used.

## 4. Experimental Procedure

In this section, a tee-joint whose geometry belongs to a special internal contour is proposed to demonstrate how the method was implemented. The core was prepared in accordance with the optimal ingredients and process parameters determined by a Taguchi method. After the product was molded, the geometrical accuracy of the product and the core removal rate of the core were measured to evaluate the proposed method. 

### 4.1. The Demonstrated Product and Its Mold Design

**Figure 3:**
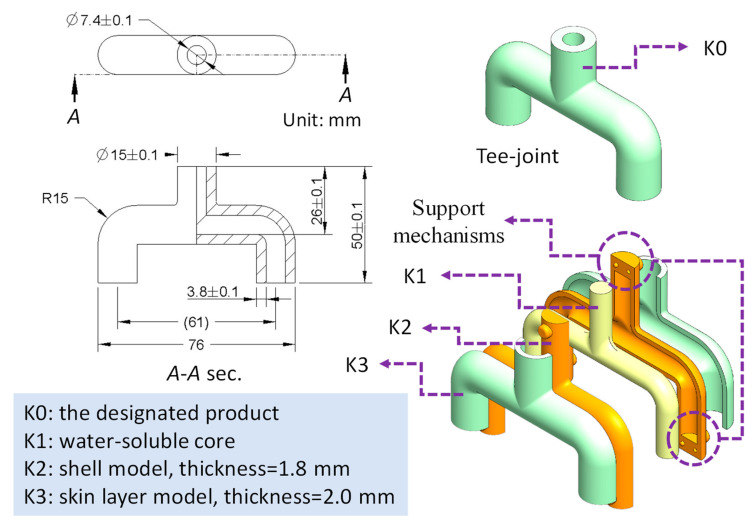
Schematic drawing of the designated tee-joint (K0), the core (K1), the shell model (K2), and skin layer model (K3).

The demonstrated product is a tee-joint (K0), as illustrated in Figure 3, whose structure is difficult to make by using only the traditional molding method. The internal contour of the tee-joint is a circle whose nominal diameter shown in the A-A sectional view is 7.4±0.1 mm. The space constructed by the assembly of two-shell model (K2) must equal the volume of the core (K1). Remember that the support mechanisms were generated and integrated at the ends of each shell model. The thickness used for the shell model and the skin layer model (K3) are 1.8 and 2.0 mm, respectively. All of them are illustrated by Figure 3 schematically. 

**Figure 4:**
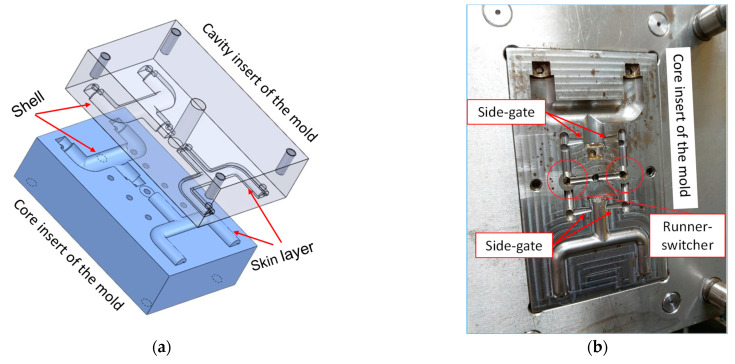
(**a**) Sketch of the core and cavity inserts, and (**b**) the half side of the test mold.

The test mold contains two cavities: one for the shell model and the other for the skin layer model. The runner-switcher was designed to change the use of each cavity individually. To reduce the core shift problem, two side-gates of each cavity were used for a balanced flow consideration. The core and the cavity inserts and the test mold are shown in Figure 4a,b.

### 4.2. Materials and Injection Molding

For a recyclable consideration, the plastic materials used for both models are the same, and acrylonitrile butadiene styrene (ABS, POLYLAC PA-757, from CHIMEI Corp., Taipei, Taiwan) was selected. To carry out a clear investigation of the interface between the shell and the skin layer, introducing pigment into the material is recommended. To minimize the effect of moisture, the ABS material was dehumidified at 80 °C for 4 h before the molding process. The molding experiments were performed on an injection machine (FANUC ROBOSHOT, S-2000i 50A, Yamanashi, Japan). To obtain the desired quality and reduce residual stress, we used two different mold temperatures, 50 °C for the shell model and 80 °C for the shell model, respectively. A higher mold temperature is beneficial for reducing the frozen thickness of the molten plastic in the mold cavity in order to improve the core deformation problem. The main injection parameters were set as listed in Table 2. 

### 4.3. Evaluation of the Molded Product (Dimensional Measurement and Core Removal Rate)

**Figure 5:**
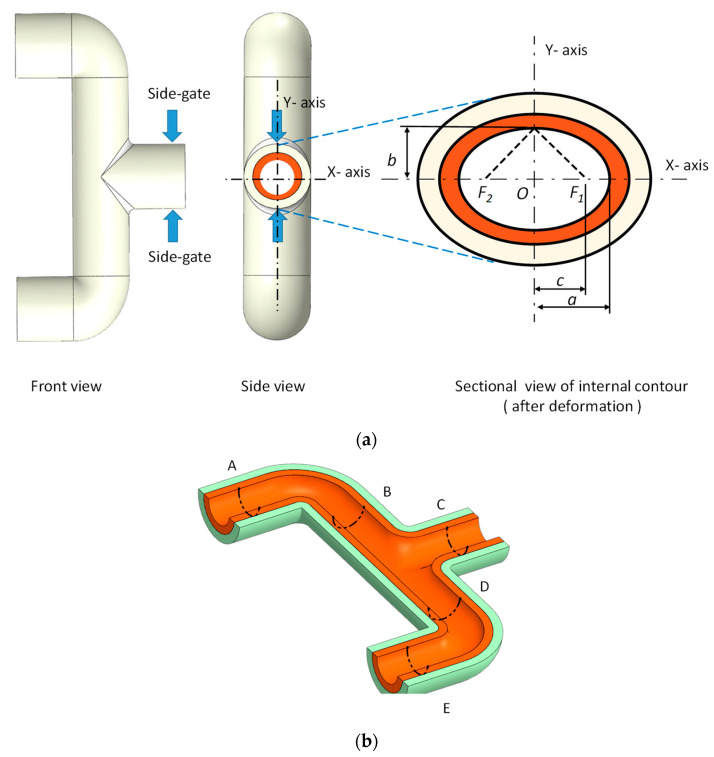
(**a**) Eccentricity measurement of the molded product and (**b**) five locations defined for each sample measurement.

Since two side-gates are used for the mold design, the internal contour deformation of the tee-joint induced by injection pressure is similar to an ellipse. Therefore, the eccentricity measurement of an ellipse is introduced to understand how much deformation of the enveloped core has occurred after the molding process has been completed. Assume that an ellipse illustrated by Figure 5a contains two axes: the major axis (longer) and the minor axis (shorter). The major and the minor radii are represented by *a* and *b*, respectively. Foci (*F*_1_ and *F*_2_) lie on the major axis and are symmetrical to the center of the ellipse. The eccentricity of an ellipse is defined by the following equation:

(2) e=c/a

where *c* is the distance between the center to the focus of the ellipse and can be calculated by applying the Pythagorean theorem:

(3) $$c=\sqrt{a^{2}-b^{2}}$$

By substituting the measured data into Equations (2) and (3), the eccentricity was calculated. Less eccentricity means that the shape of the measured specimen is nearly a perfect circle. The sample was cut into two parts symmetrically, as shown in Figure 5b, to measure the internal contour on the five locations. In each location, select four points averagely for circumference measurement; therefore, the major radius, *a*, and the minor radius, *b*, were obtained automatically by using the coordinate measuring machine (Brown & Sharpe Co., Ltd., Inspector classic, North Kingston, RI, USA). 

**Figure 6:**
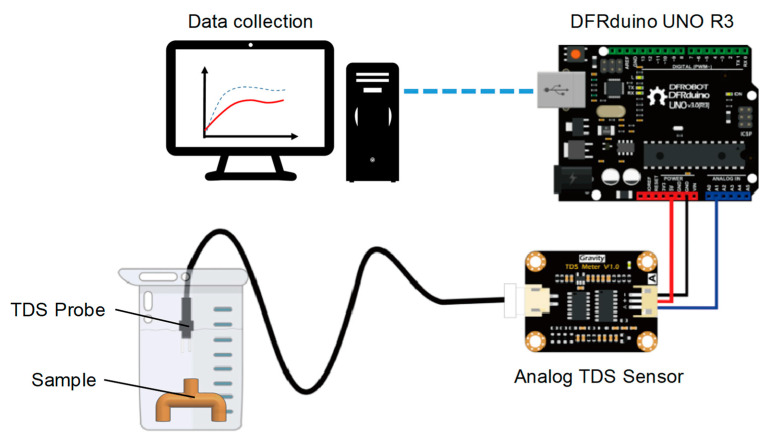
The schematic illustration of the core removal rate measurement.

The core removal rate is also an important indicator of the productivity for core-losing methods. When salt dissolves in water, the ions from the salt increase the conductivity of the solution. To acquire quantitative information on the core removal rate, the conductivity of the water bath in which the sample is immersed is measured. The facility of core removal rate measurement shown in Figure 6 includes a conductivity meter (DFRobot SEN0244), TDS (total dissolved solids) probe, and a data acquisition board (DFRduino UNO R3) for data collection. For stable measurement, the beaker was put on a shaker with a vibration frequency of 20 Hz for 2 min. A baseline was created by measuring the conductivity of known quantities of NaCl in one liter of distilled water at 30 °C in advance. Each test sample was then put in the beaker with the same operation procedures. The conductivity of the solution in the beaker was recorded with respect to the time and transformed into the core removal rate. The core removal rate, ε, is expressed as follows:

(4) ε=ρρ0×100%

where *ρ*_0_ and *ρ* are the conductivities of the baseline and the test sample, respectively. A higher value of ε indicates that much filler of the test sample was dissolved.

## 5. Results and Discussion

A Taguchi method used to evaluate the different parameter combinations on the core strength was first executed. After the optimal parameter was determined, the proposed sample, a tee-joint, demonstrated how the proposed method can be implemented. Both the dimensional accuracy of the molded product and the core removal rate were investigated in the second and third examples.

### 5.1. Analyses of S/N and ANOVA Results 

**Figure 7:**
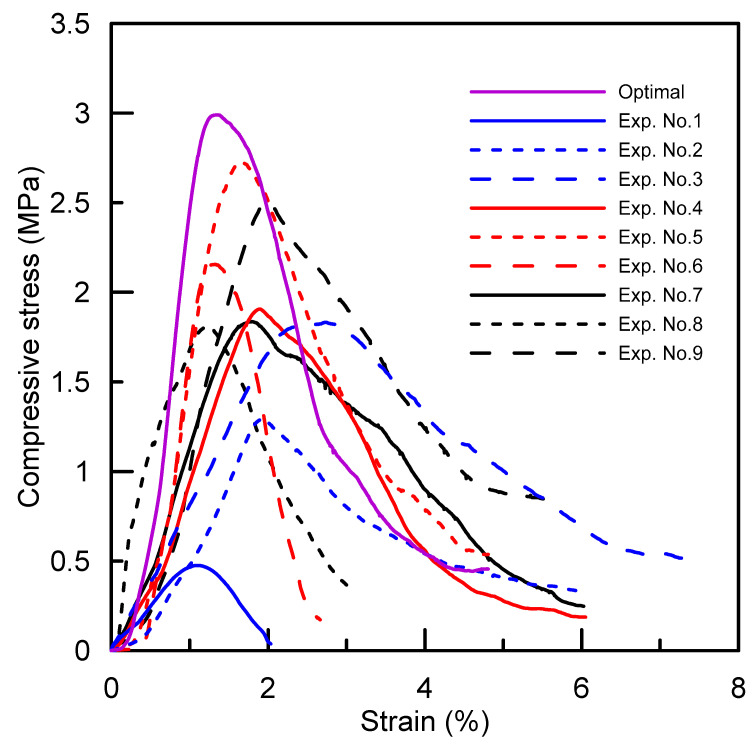
Compressive-stress-and-strain curves for salt core with respect to setting parameters.

The compressive-strength results of the specimen prepared by the setting parameters of the orthogonal array *L*_9_ are listed in Table 3. The analyses of the *S*/*N* ratio and the variance (ANOVA) provide further information, such as the following: the degrees of freedom (*DF*), sequential sums of squares (*SS*), mean square (*MS*), the *F*-statistic (*F*), etc., to figure out if the results are significant or not. The ranks of all the factors are sorted by the delta (∆), which is defined by the range between the levels change of the factors. A larger value of ∆ means that the contribution from the factor to the compressive strength is more effective. The ANOVA results listed in Table 4 were calculated from the test results, as shown in Table 3, and used to explore the effects of parameters on the compressive strength of the specimen. Therefore, the optimal parameters combination, *A*_3_*B*_3_*C*_2_*D*_3_, was suggested, which does not appear in the orthogonal array *L*_9_. Moreover, the ANOVA results indicate that the binder content and the applied pressure have a significant effect on the compression strength: their contribution percentages are 36.3% and 26.7%, respectively. Furthermore, a verification test was also conducted and compared with the compressive strength of the nine design experiments. The results are shown in Figure 7: the compressive strength of the specimen prepared by using the optimal parameters is 3.2 MPa, and it exhibits a 17.2% improvement compared to the maximum strength of Exp. No. 5 listed in Table 3.

### 5.2. Dimensional Measurement of the Molded Product

**Figure 8:**
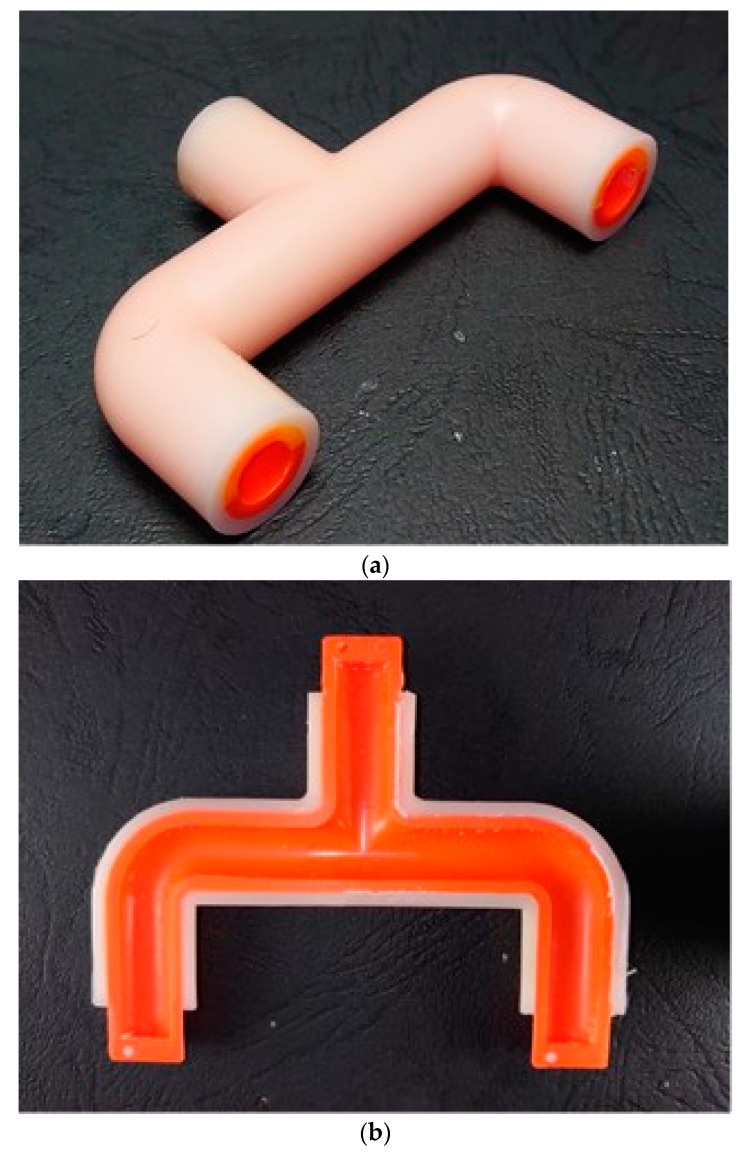

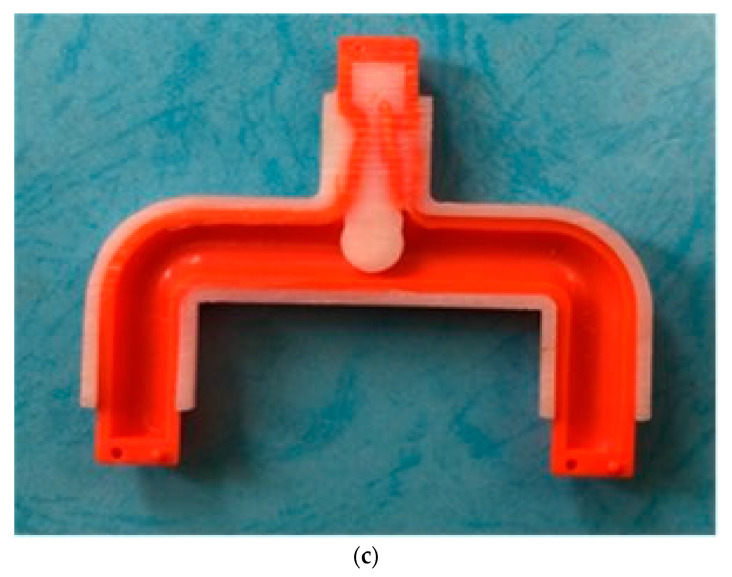
Photo of (**a**) the designated product, tee-joint; (**b**) sectional view of the sample made by optimal parameters, *A*_3_*B*_3_*C*_2_*D*_3_; and (**c**) sectional view of the sample made by setting parameters, *A*_2_*B*_1_*C*_2_*D*_3_.

**Figure 9:**
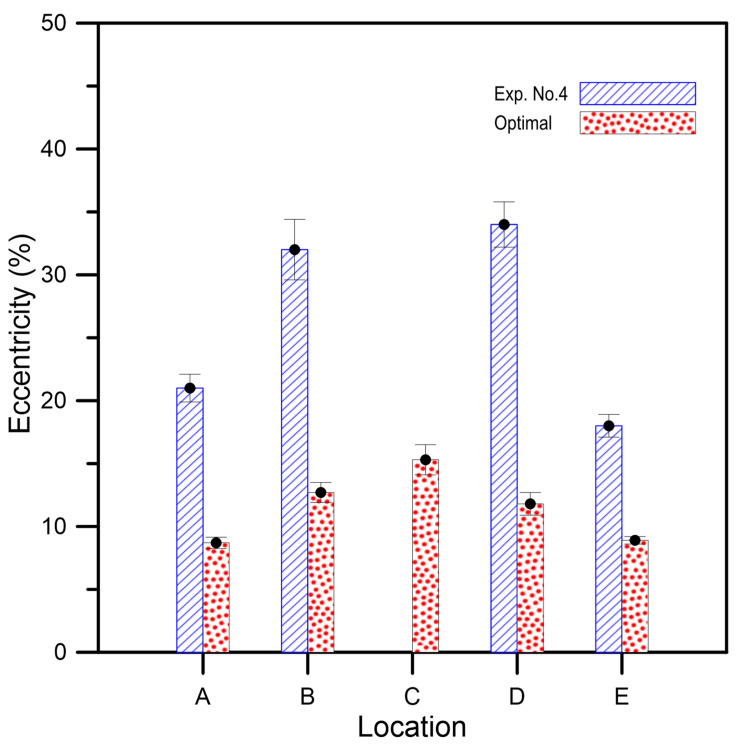
The eccentricity measurement of internal contour of samples.

This example demonstrates the influence of the core prepared by different setting parameters on the dimensional accuracy of the tee-joint. The molded product shown in Figure 8a is a tee-joint sample made by the optimal parameter suggested in the previous example. The sectional view shown in Figure 8b is the sample molded by using the optimal parameters, *A*_3_*B*_3_*C*_2_*D*_3_, and its internal contour is smooth. Although the thickness of the shell layer is close to that of the skin layer, the impacts of pressure and temperature induced from the molten plastic onto the shell are extremely large. As a result, the deflection of the shell causes the molten plastic to flood into the core, and the product fails. When the compressive strength of the core is less than 2.0 Mpa, e.g., prepared by Exp. Nos. 1, 2, 3, 4, 7, and 8 of Table 3, an insufficient strength of the core cannot resist the melt impact from deformation. The internal contour of the destroyed sample shown in Figure 8c was made by the parameter combination of *A*_2_*B*_1_*C*_2_*D*_3_ (Exp. No. 4). Furthermore, the eccentricity measurement of both samples on five locations is shown in Figure 9. Except for location *C* of the destroyed sample, the five eccentricities of the optimal sample are less than those of the destroyed sample. The average eccentricity of the sample made by the optimal parameters is 44% lower than that of the destroyed sample.

### 5.3. Core Removal Rate of Water-Soluble Core

**Figure 10:**
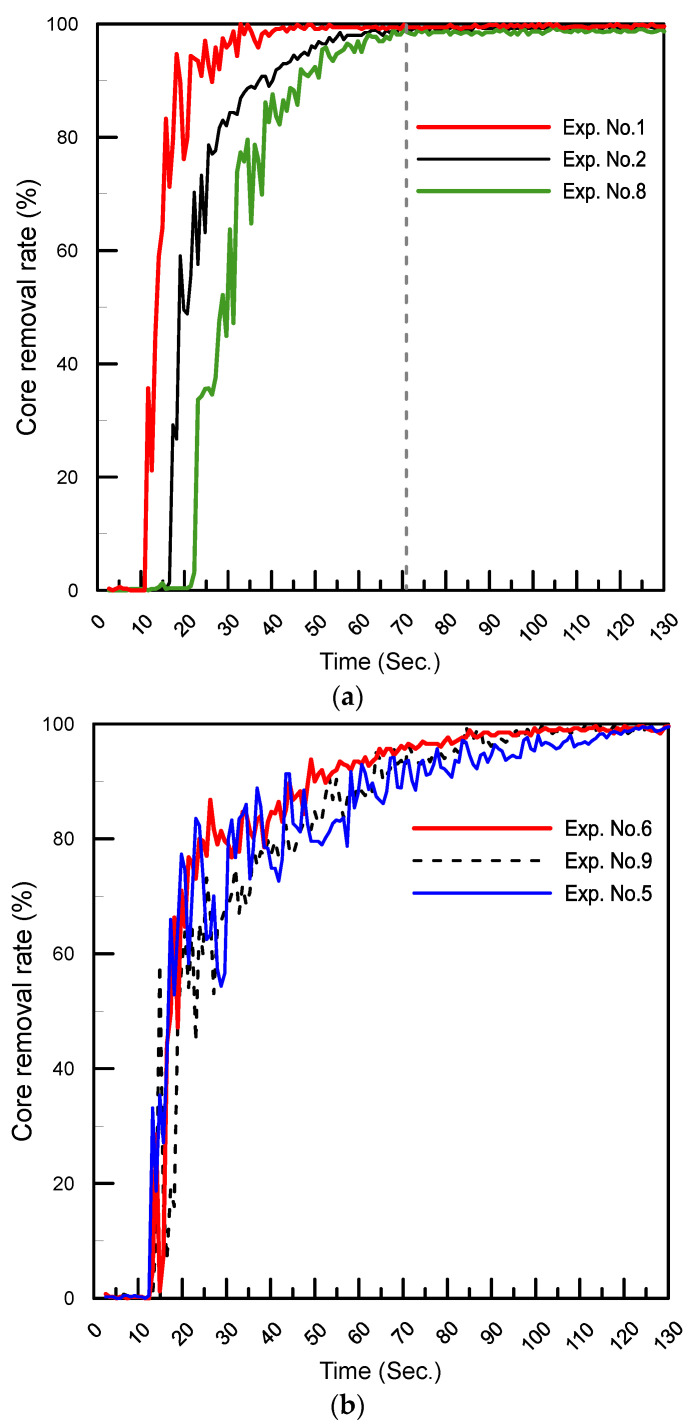
(**a**) Core removal rate of the lowest strength group and (**b**) core removal rate of the highest strength group.

In this example, the cores made of the different parameter combinations of Table 3 can be ranked and divided into three groups according to the compressive strength performance. Since the core removal rate of the salt core was decreased as the compressive strength of the core was increased [11], we selected the highest strength and the lowest strength groups for the core-removal rate test; the results are shown in Figure 10a,b, respectively. The curves of the core removal rate of the lowest strength group were saturated at 70 s, meaning that the core was dissolved completely after 70 s. Compared to those curves of the highest strength group, the saturated time was extended to 130 s, proving that the stiffer part of the core needs more time for the core to dissolve. The core made by using the optimal parameters was also tested, and the required time for the core to dissolve was 154 s.

## 6. Conclusions

The concept of the enveloped core was developed to enable a special hollow product to be manufactured by injection molding without any assembly process. The enveloped core provides both rigidity and toughness to prevent the molded product from experiencing deformation. The cooperation of the salt core and the shell extends the application of the traditional water-soluble core from a low-pressure molding process to a high-pressure molding application. The results indicate that the binder content and the applied pressure have a significant effect on the compression strength. The internal contour of the demonstrated product, a tee-joint, is smooth, and its eccentricity measurement is improved from 26.3% to 11.5%. The filler (salt) and binder (sugar) are readily available and inexpensive and can be quickly dissolved in water. There is also the potential to recover some salt or sugar from the waste solution. Compared with the fusible-core injection molding method, the proposed method provides advantages, including lower energy consumption, easy to implementation, and no limitation of resin type. The utility of the proposed technique conveniently enables the product with a special internal contour to be accomplished.

## Figures and Tables

**Table 1:** Parameters and associated levels in the Taguchi study.

	Level 1	Level 2	Level 3
*A*, Binder content (wt.%)	5	8	12
*B*, Applied pressure (MPa)	3	6	9
*C*, Processing time (min)	30	45	60
*D*, Processing temperature (°C)	120	150	190

**Table 2:** Molding parameters for the shell and the skin layer model.

	Shell Model	Skin Layer Model
Barrel temperature, °C (from nozzle to feed zone)	200–210–200–190	200–210–200–190
Injection speed, mm/s	110	120
Mold temperature, °C	50	80
Cooling time, s	22	35
Holding pressure, bar	200	250
Holding time, s	2	3.2

**Table 3:** Experimental and S/N results of compressive strength.

Exp. No.	Setting Parameters	Compressive Strength	
		Exp. Results (Mpa)	S/N (dB)
1	*A*_1_ *B*_1_ *C*_1_ *D*_1_	0.48	−8.29
2	*A*_1_ *B*_2_ *C*_2_ *D*_2_	1.29	1.29
3	*A*_1_ *B*_3_ *C*_3_ *D*_3_	1.83	4.61
4	*A*_2_ *B*_1_ *C*_2_ *D*_3_	1.90	5.36
5	*A*_2_ *B*_2_ *C*_3_ *D*_1_	2.73	8.37
6	*A*_2_ *B*_3_ *C*_1_ *D*_2_	2.15	5.98
7	*A*_3_ *B*_1_ *C*_3_ *D*_2_	1.83	4.69
8	*A*_3_ *B*_2_ *C*_1_ *D*_3_	1.81	4.64
9	*A*_3_ *B*_3_ *C*_2_ *D*_1_	2.5	12.14

**Table 4:** Summary of ANOVA results for maximum compressive strength.

Factor	DOF	Average S/N Values			∆	*SS*	*MS*	*F*	*P* (%)
		Level 1	Level 2	Level 3					
*A*, Binder content	2	−0.79	6.57	7.15	7.94	494.7	247.3	1.19	36.3
*B*, Applied pressure	2	0.59	4.77	7.57	6.98	364.1	182.1	0.87	26.7
*C*, Processing time	2	0.78	6.26	5.89	5.48	310.9	155.4	0.75	22.8
*D*, Processing temperature	2	4.07	3.99	4.87	0.88	146.3	73.2	0.35	10.7
Error	18	−	−	−	−	417.1	23.2	−	3.40
Total	26	−	−	−	−	1733	−	−	100

## Data Availability

The data presented in this study are available on request from the corresponding author.
